# Implantable Cardiac Pacing Devices Related Complications: Keeping Pace With Time

**Published:** 2011-02-08

**Authors:** Shomu Bohora

**Affiliations:** Assistant Professor, U.N. Mehta Institute for Cardiology and Research Centre, Civil Hospital Campus, Ahmedabad, India

**Keywords:** Cardiac Pacing Devices, Complications

*The physician should look upon the patient as a besieged city and try to rescue him with every means that art and science place at his command.*  ~ Alexander of Tralles.

Science of medicine (and less of art) has progressed tremendously over the last few decades and engineering innovations have constantly changed the way we used to treat a patient. The field of cardiology is the best example of how technology has invaded into a human body making interventions a lot easier and safer. Cardiac pacing devices which initially were designed to treat bradycardia, have now found place for treatment for ventricular arrhythmia, heart failure and for prevention of sudden cardiac arrest. With increasing number of patients having cardiac disease and with the ever expanding indications of device therapy in clinical practice, the numbers of patients who are undergoing device therapy is expanding exponentially.

Based on knowledge of inadequacies from prior implant data, cardiac pacing devices and related hardware are constantly undergoing refinement and are continuously being upgraded to being better and safer each time. Lead is placed in the right atrial appendage for atrial pacing and right ventricular apex or septum for right ventricular pacing for most of the patients through the axillary/subclavian/cephalic venous access. The pulse generator is kept most commonly in the subcutaneous or submuscular pocket in the pectoral region.  Active fixation lead (screw in lead) or passive fixation (tined) leads are selected based on patient's disease, need to do special site pacing or purely on operator preference. Pacing in the right atrial free wall, interatrial septum, right ventricular outflow, His bundle and left ventricle requires greater understanding of anatomy and/or training in using appropriate hardware and techniques for appropriate lead placement. Occasionally surgeons help needs to be taken for epicardial lead implantations. [1-3]

Though implantation of cardiac pacing device is now a safe time tested procedure, complications related to implantation, when enumerated are many, but occur in about 5.7% of patients and can be grouped as either procedural, component or biophysical interface related problems.[1-3] Intuitively and scientifically complications are more likely to occur with increased procedure time, more difficult procedure or implantation technique (like upgrade of existing devices or left ventricular lead implantation), with implantations in higher risk patients and with lesser operator experience.[2] Though, once in a while, complications do occur in hands of even the most experienced operator in the most simple device implantation procedure and in an absolutely normal risk patient. Precautions hence need to be taken appropriately and hardware selection always needs to be individualized.

Commonly occurring complications of percutaneous venous access and blind subclavian puncture are subclavian artery puncture, pnuemothorax, hemothorax and hemo-pneumothorax. Lacerations of subclavian artery, nerve injury, thoracic duct injury, chylothorax and lymphatic fistula have occasionally been described. Contrast venography-guided venipuncture, ultrasound-guided puncture and an extra-thoracic subclavian puncture may decrease such complications and fluoroscopy time.[4] Though cephalic venous cut-down has decreased such vascular and pleural complications, multiple lead insertions cannot be achieved with cephalic venous cut-down alone and hence venous access related complications occasionally do continue to occur.[5,6]

Acute complications of lead placement include thromboembolism (air/clot), arrhythmia, tricuspid regurgitation due to damage to the valvular apparatus and chamber perforation associated with or without cardiac tamponade. Left ventricular lead placement presents special challenges and complications due to need for coronary sinus cannulation and placement of a lead in a desired vein. Lead displacement with rise in thresholds, loss of pacing, diaphragmatic pacing and chamber perforation, pericarditis with or without cardiac tamponade can occur either immediately or at a later date. Misplacement of a lead is very uncommon, though described (lead placed in a left ventricle through the interatrial septum instead of desired right ventricle pacing). [2,4,7]

Device related complications include battery failure and pulse generator circuit failure, lead failure, conductor coil fracture and insulation failure. [3] Manufacturing deficiencies in software or hardware have rarely led to device/lead recalls. Under-sensing, over-sensing and programming related issues leading to inappropriate therapy tend to crop up every now and then and most of the time can be appropriately rectified non-invasively. Electromagnetic interference may occasionally cause device malfunction.

Axillary vein thrombosis is rare occurring in 0.5-1% of cases. Partial venous obstruction in the great veins is almost a rule and occurs to some degree in up to 100% of cases. Clinically, pulmonary embolism however is extremely rare. Partial or silent inconsequential thrombosis is considered extremely common but generally of no clinical significance. [1,4] Pain at the local site and shoulder pain can sometimes be annoying.

Pocket related complications like pocket hematoma, wound dehiscence, migration, erosion, pain and infection are well known and almost all who perform the procedure routinely have come across varying severity of such complications. [3] Twiddler's syndrome is very uncommon. [8] Device related infections possibly present the greatest challenges in clinical practice. Infections may present acutely with septicemia with or without endocarditis with vegetation on the lead, valve or the cardiac tissue or a pocket abscess. Chronic infection most often presents as a chronically discharging sinus, device erosion or a granulomatous mass. [3] Explantation of the whole system and reimplantation at a different site or reimplantation after adequate debridement and antibiotic therapy at the same site are almost always required in either acute or chronic settings and requires patience and persistence of both the patient and the treating physician. Extraction of chronically implanted leads can be challenging and can be associated with significant complications. [3]

If not vigilant, recognition of uncommon complications like a contralateral pneumothorax due to a right atrial micro-perforation by an atrial screw in lead described by Syamkumar et al in the issue of this journal sometimes can get delayed.[9] Delayed perforation leading to migration of the lead to the pericardium or the pleural cavity with or without pericarditis, cardiac tamponade or hemothorax have been described.[10-14] Such complications though very uncommon are potentially life threatening and should be recognized and treated immediately.

Follow up of patients needs to be done regularly with cardiac devices and should be emphasized in all patients who receive them. It is not only necessary for optimizing battery life, but also to detect complications early related to the device or the biophysical interface so that correction can be done before significant symptoms develop. The case report by Garg et al presents an interesting patient, who after initial implantation never came for follow up, and presented with end of life of pulse generator and a chronic granulomatous mass over the incision site described due to hypersensitivity to non-absorbable suture material. [15] Ideally chronic infection should be considered in all such patients and further treatment should be done accordingly. [3]

Atypical complications can occur. Complications though unavoidable, its manifestations can be minimized by early recognition and appropriate treatment. The absolute number of complications that we may see in the near future is going to increase, though the percentage of complications decrease with increase in the operator experience and better hardware, as absolute number of implantations occurring are increasing. Till the day, when advances in bio-technology would change the way we implant cardiac pacing devices for treatment of bradycardia, heart failure, ventricular tachycardia and for prevention of sudden cardiac arrest, we may still continue to face complications related to the interventions we do.
